# Clinical Cases

**Published:** 1895-10

**Authors:** A. L. Kennedy

**Affiliations:** Boston, Mass.


### SOCIETY OF HOMŒOPATHICIANS.

### Meeting of 1895.

### Second Day. Morning Session.

Thursday, June 27th, 10.15 A. M.

### Bureau of Clinical Medicine.

E. W. SAWYER, M. D., CHAIRMAN.

### CLINICAL CASES.

A. L. Kennedy, M. D., Boston, Mass.

### Case I.

In August, 1894, Mr. J. consulted me for slight dullness of hearing of the right ear accompanied by a sense of fullness and some roaring and humming in the head. Three weeks previously while bathing he had got water in his ears and had been troubled ever since.

Mr. J. has now and then consulted me, at which times he has usually complained only of those symptoms that naturally arise as the result of a cold; though now and then he had spoken of urinary symptoms of somewhat troublesome character, and two or three times had complained of seminal weakness amounting to almost inability for sexual intercourse. There was a history of syphilis, but his general health was good, and with the above exception and now and then a few "twinges of rheumatism," he managed to get along very comfortably.

I had prescribed for him from time to time as the symptoms indicated, and he would be relieved for the time being, and until a fresh cold or other exciting cause again awakened new symptoms.

On the occasion of his visit above referred to I prescribed as usual, and also at one or two subsequent visits, but with very indefinite results. His condition remained much the same.

At length on the 23d of November he again consulted me, after an absence of more than two months. His statement was as follows: Had received treatment for his ear (having consulted an aurist). Got relief of the trouble with the ear but since that time his eyes had troubled him. He complained also of a "dull, tired ache" in the legs day and night, but worse at night. Headache through the temples, coming and going, day and night. Severe pain at times in the cardiac region, also at a corresponding point on the right side. Must rise two or three times at night to urinate; must wait for urine to start. Urine voided more easily on standing; dribbles at times.

Appetite poor, thirst, constipation, mouth dry, pulse 90. A few tiny blotches of a reddish hue had appeared on the face. The latter wore an anxious expression, and althogether the man appeared decidedly ill. He had been using *Ka-iod.*, *Prot.*, *Iod. of Merc.*, and *Lithia-carb.*, all in appreciable doses. I gave *Hepar.*

Five days later he came again. He now complained of inability to sleep, owing to the pains. Sharp pains in both temples and especially around and in the eyes, and passing back toward the neck—at one time worse in the left side. For some time has had a peculiar feeling in the legs, on attempting to run, a sort of "griping" as of a "cramp." Upon attempting to walk rapidly will have a feeling that he cannot do it; must stop; legs feel at such times "as if in a vice." Pains worse *in bed* and at *rest.*

Better moving about or from gentle exercise. Head worse at night on lying. Urine slow in starting and voided much easier on standing. Frequent calls to urinate. Troubled dreams. *Thinks legs are tied together and won't work.* Dreams also of business perplexities. Syphilin.$^{m}$ (F.).

December 4th, one week, reports: Head all right. Eyes still trouble some. Aching in legs better. Has had two or three good nights. A little "lightness" in legs. Calls to urinate less frequent. No dreams to trouble. Appetite improved. Placebo.

December 12th (eight days).—Has been steadily gaining. He called upon me a short time ago for some slight ailment, and told me he was feeling finely. In fact, he looked it. He had good color, had gained in weight, and had the appearance of being in every way a well man.

### Case II.

About two years ago I was called to see a young woman who had been strongly advised to submit to an operation for the removal of the ovaries.

As nearly as I could judge from the history that she gave, she had suffered from inflammation of the right ovary and also from inflammation of the pelvic cellular tissue. Her general health was fair, though repeated attacks of pain with swelling, followed by vaginal discharges of a bloody, purulent character, and accompanied by more or less elevation of temperature, had finally had its effect, and she felt that her strength was beginning to falter.

She had been repeatedly told by her attending surgeon that an operation was the only means that could afford her any satisfactory or permanent relief.

She wished to leave the city for the summer, but was told that such a step would be attended by great risk, as at any time she was likely to require the services of the surgeon, that he might operate immediately in order to save her life.

In conversation with the patient I found that while she was pleased at the idea of possible relief without resorting to surgery, yet she was evidently skeptical as to the result in the use of medicines alone; however, after a little talk, she consented to receive treatment from me, and I proceeded to make a record of her case with a view to carrying out her wish.

After a little time the patient asked that she might go out-of-town. As she seemed so anxious to make this change in her surroundings, and I could not see that there was any likelihood of serious results by her removal, I told her she might go.

She accordingly went to Rhode Island and spent the summer. From time to time she suffered from the recurrence of her former symptoms, but with gradually decreasing severity, so that upon her return in the fall there was evidence of improvement in her general condition, and she had *not* been obliged to call a surgeon to save her life.

She continued under my care, receiving remedies from time to time, for perhaps more than a year before she was entirely relieved of the trouble that so long had annoyed her, and at one time assumed so formidable an appearance. For some months now she has been the picture of health, and this notwithstanding the fact that her mother has been ill, and she has taken care of her, beside doing the housework for the family, necessitating a good deal of going up and down-stairs.

But two or three days ago I received a letter from her, in which she stated that she seldom has an ache or a pain, and were it not for a tired feeling that she has at times (owing, doubtless, to the hard work she has done) she could consider herself *well.*

